# Post-Mortem Examination of a Cryptorchid Bullock

**Published:** 1901-06

**Authors:** C. H. Sweetapple

**Affiliations:** Professor of Cattle Pathology and Obstetrics, Ontario Veterinary College


### REPORTS OF CASES.

### POST-MORTEM EXAMINATION OF A CRYPTORCHID BULLOCK. CONGENITAL MALFORMATIONS AND PATHOLOGICAL CONDITIONS.

C. H. SWEETAPPLE, V.S.,
PROFESSOR OF CATTLE PATHOLOGY AND OBSTETRICS, ONTARIO VETERINARY COLLEGE.

"IN life that vegetates, and life that moves, o'er all disease her beauty-withering wand waves high," and it cannot be doubted that in all "life that moves" congenital malformations may also frequently exist, perhaps unsuspected.

The following case, from notes of a post-mortem examination made for the Health Department of the city of Toronto a few weeks ago, will, I trust, prove of interest :

A white steer, about three years old, had a masculine (staggish) appearance about the head, and was in good condition and apparent good health. In consequence of having a tumor in the soft tissues in the region of the throat, the animal was held under the authority of the City Health Department to be butchered under veterinary inspection. The bullock was killed and the viscera removed by the butcher in the ordinary way. On opening up the animal it proved to be a cryptorchid, a small, imperfectly developed testicle being found in the posterior inferior parts of the abdominal cavity attached to the spermatic cord. This, of course, accounted for the staggish appearance of the animal. The liver was normal in size and form, but had two gall-bladders closely united together. The butcher told me that he had occasionally noticed similar malformations in slaughtered animals.

On removing the stomachs an abscess was found adjacent to the reticulum. It contained a quantity of pus and a piece of steel wire, apparently about two inches of a knitting-needle.

The tumor in the throat was about the size of a large orange. It consisted of dense fibrous tissue, in the centre of which was a cavity containing a pus-like fluid. This cavity had several chambers communicating with each other. It was evidently tubercular. On the serous coat of the rumen there were a number of small tubercles in the gray or early stage of development ; also some in a similar stage on the parietal peritoneum. In the greater part of the tissue of the lungs were numerous tubercular nodules, varying in size from that of a marble to a walnut. These were in the caseous stage. There were no adhesions or tubercular nodules on either the visceral or parietal pleura. There was a tumor about as large as a duck's egg near the distal end of the fleshy portion of the gastrocnemii muscles, situated in the areolar tissue between the muscles. The muscular tissue itself was not involved. This was evidently tubercular—an enlarged lymphatic gland.

To find all these different conditions in the one animal was certainly unusual and worth recording.

